# Dietary Patterns and Their Association With Body Fat Percentage Among Ready‐Made Garment Workers in Dhaka and Chattogram, Bangladesh

**DOI:** 10.1002/puh2.70195

**Published:** 2026-02-13

**Authors:** Md. Shahadoth Hossain, Md. Hafizul Islam, Rafid Hassan, Md. Mahbub Alam, G. M. Reza Sumon, Md. Ruhul Amin

**Affiliations:** ^1^ Institute of Nutrition and Food Science University of Dhaka Dhaka Bangladesh; ^2^ Department of Nutrition and Food Engineering Daffodil International University Dhaka Bangladesh; ^3^ Nutrition Research Division International Centre for Diarrhoeal Disease Research Bangladesh (icddr,b) Dhaka Bangladesh; ^4^ Global Alliance for Improved Nutrition (GAIN) Dhaka Bangladesh

**Keywords:** Bangladesh, body fat percentage, dietary habits, dietary patterns, nutritional status, ready‐made garment workers

## Abstract

**Background:**

The ready‐made garment (RMG) sector plays a critical role in Bangladesh's economy, contributing substantially to both GDP and employment. This study investigated the dietary patterns of RMG workers in Bangladesh and explored their association with percent body fat.

**Methods:**

A cross‐sectional study was conducted among 576 RMG workers from three factories in the Dhaka and Chattogram regions. Dietary intake was assessed using the 24‐h recall method. Principal component analysis was used to identify major dietary patterns. Diet quality was measured using the nutrient adequacy ratio (NAR) and mean adequacy ratio (MAR). Body fat percentage was assessed using bioelectrical impedance analysis, and body mass index was classified according to Asian‐specific cut‐off values. Logistic regression models were used to examine the associations between dietary patterns and body fat percentage.

**Results:**

Over half of RMG workers (51.2%) were overweight or obese, and 47.2% exhibited elevated body fat. Carbohydrates were the primary source of energy, contributing 67.7% of total intake, whereas inadequacies in several essential micronutrients, particularly calcium, vitamin A, and riboflavin, were observed, with the MAR for 11 micronutrients at 72%. Five predominant dietary patterns were identified across the two regions. Notably, in the Chattogram region, workers who adhered more closely to the “dairy‐based festival diet” (a pattern rich in milk‐ and sugar‐based festive foods) exhibited a significantly higher body fat level (adjusted odds ratio [AOR] = 2.44, 95% confidence interval [CI]: 1.41, 5.75; *p* = 0.040) compared to others.

**Conclusion:**

This study reveals that carbohydrates dominate energy intake alongside widespread micronutrient inadequacies among RMG workers. Greater adherence to a dairy‐based festival diet was associated with increased body fat. Targeted nutritional interventions are needed to improve diet quality and reduce obesity‐related risks.

## Graphical abstract

Ready‐made garment workers in Bangladesh show high rates of overweight, elevated body fat, and micronutrient deficiencies, linked to carbohydrate‐ and festive food–rich diets, highlighting the need for targeted workplace nutrition interventions to improve diet quality and reduce obesity‐related health risks.

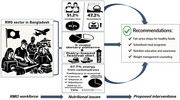

## 1 Introduction

Body fat plays a crucial role in overall health, serving as an energy reservoir and contributing to various physiological functions. However, excessive body fat, particularly visceral fat, is a well‐established risk factor for numerous chronic diseases, including cardiovascular diseases (CVDs), type 2 diabetes, hypertension, and metabolic syndrome [1, 2]. In contrast, insufficient body fat levels can lead to nutritional deficiencies, weakened immunity, and impaired physiological functions [3, 4]. The global prevalence of overweight and obesity has reached alarming levels, with over 2.5 billion adults classified as overweight and 890 million as obese in 2022 [5]. This increasing adiposity is a major contributor to the burden of noncommunicable diseases (NCDs), which accounted for approximately 75% of non‐pandemic‐related deaths worldwide in 2021 [6]. Among these, CVDs alone were responsible for an estimated 17.9 million deaths in 2019, comprising 32% of all global deaths, with heart attacks and strokes accounting for 85% of these fatalities [7].

Diet plays a pivotal role in determining body composition, influencing fat accumulation and distribution [8]. Body fat accumulation is influenced not only by total calorie intake but also by the quality of the diet [9]. Specific dietary patterns can have significant effects on fat distribution, metabolism, and inflammation [10]. Unhealthy diets characterized by high levels of processed foods, refined carbohydrates, and trans fats are strongly associated with increased visceral fat and the onset of metabolic disorders such as hypertension, diabetes, and CVD [11, 12]. Conversely, diets rich in fiber, fruits, vegetables, and lean proteins contribute to lower body fat percentages by promoting satiety and enhancing metabolic function [13, 14]. Notably, adherence to the Mediterranean diet, which emphasizes healthy fats, whole grains, and plant‐based foods, has been linked to reductions in both abdominal and overall body fat [15].

Bangladesh, like many low‐ and middle‐income countries (LMICs), is undergoing rapid urbanization and economic development, leading to significant shifts in dietary habits and lifestyle behaviors [16]. This transition has contributed to a dual burden of malnutrition, where undernutrition persists alongside a rising prevalence of overweight and obesity [17, 18]. According to the 2022 Bangladesh Demographic and Health Survey (BDHS), 20.1% of adult men were overweight or obese [19]. Similarly, among women of reproductive age, 55.5% were overweight or obese, with a notably higher prevalence in urban areas [20]. In addition to the rising prevalence of overweight and obesity, other NCDs are also widespread in Bangladesh. A study utilizing data from BDHS 2017–18 revealed that 28.01% of the adult population suffers from hypertension, whereas 10.27% are affected by diabetes mellitus [21]. Furthermore, findings from the WHO STEPwise survey conducted in 2018 indicate that more than half of the respondents do not meet the criteria for ideal cardiovascular health [22].

Obesity‐related chronic diseases not only impose a substantial economic burden on the healthcare system but also reduce workforce productivity [23, 24]. The ready‐made garment (RMG) sector plays a crucial role in Bangladesh's economy, contributing significantly to GDP and employment [25]. The majority of RMG workers, predominantly women from rural areas, experience demanding work conditions, low wages, and limited access to nutritious foods, making them vulnerable to poor dietary habits and inadequate nutritional intake [26]. This vulnerability is evident in studies reporting that 23% of RMG workers are overweight and 27.9% are obese, alongside a high prevalence of hypertension (14.5% prehypertensive and 19.5% hypertensive) and diabetes (7.3% diabetic and 10.6% prediabetic) [27]. These health challenges are exacerbated by poor dietary diversity, inadequate healthcare access, and suboptimal nutritional awareness [28]. Given the economic significance of the RMG workforce, addressing their dietary and nutritional challenges is vital to enhancing their health and productivity.

Dietary pattern analysis offers several advantages over traditional single‐nutrient approaches in nutritional epidemiology [29]. Traditional approaches focusing on single nutrients often fail to account for the interactions between different dietary components and their cumulative effects on health outcomes [30]. Dietary pattern analysis, on the other hand, provides a more comprehensive perspective by capturing habitual eating behaviors and overall dietary quality. This approach aligns with current global dietary recommendations, emphasizing the importance of whole‐food consumption rather than isolated nutrients [31]. This approach has been widely used to assess chronic disease risk, and in Bangladesh, principal component analysis (PCA) has been employed to identify dietary patterns associated with heart disease [32], CVDs [33], hypertension [34], diabetes [35], and skin lesions [36]. These findings highlight the importance of evaluating dietary patterns rather than individual nutrients to better understand their role in body fat accumulation and metabolic health.

Existing studies on the dietary habits of RMG workers focused primarily on their typical food consumption [26, 37], yet there is a lack of clear understanding regarding their dietary patterns and nutrient intake levels. Although few studies have examined the nutritional status of RMG workers by assessing their body mass index (BMI) [27, 28, 38], there is a complete lack of research on the body composition of this population. Furthermore, no studies have explored how dietary patterns influence body fat percentage among RMG workers, despite their critical role in Bangladesh's economy and their vulnerability to poor dietary habits. To address this critical gap, this study aimed to assess the dietary patterns and their association with percent body fat among garment workers in Bangladesh, thereby generating evidence to inform targeted nutritional interventions.

## 2 Materials and Methods

### 2.1 Study Design and Setting

A cross‐sectional study was conducted from May 26 to June 10, 2023. A quantitative study design was implemented to collect data from workers of three selected RMG factories located in Dhamrai, Dhaka, and Patenga, Chattogram, two prominent centers of the RMG sector in Bangladesh.

### 2.2 Sample Size and Sampling Strategy

In the present study, the participants were selected through convenience sampling. The sample size was calculated using the formula for a cross‐sectional study design [*n* = *Z* ^2^ × *p*(1 − *p*)/*d* ^2^]. The value for p is obtained from the prevalence of obesity (50%) among garment workers [27]. We have considered a factor of 1.5 to multiply the estimated sample size, as the participants were not selected through simple random sampling. A total of 576 study participants were conveniently selected. Among them, 346 were from the Dhaka region, and 230 were from the Chattogram region. Overall, 225 of the participants were male, and 351 were female. As Dhaka is the largest hub of the RMG sector and women make up the majority of its workforce, our study prioritized a larger sample from Dhaka and included more female participants.

### 2.3 Data Collection

Data were collected using a validated, structured questionnaire that included modules on sociodemographic characteristics, dietary intake, and anthropometric measurements. The questionnaire was translated into Bangla and back‐translated to ensure linguistic and conceptual accuracy. Sociodemographic variables included age, sex, educational attainment, living status, household income, and expenditure. Dietary intake was assessed using the 24‐h dietary recall method. Anthropometric measurements comprised height and weight, which were obtained using standardized procedures and calibrated instruments. Trained enumerators conducted face‐to‐face interviews after receiving comprehensive training on data collection protocols, ethical considerations, and anthropometric measurement techniques. Field supervisors reviewed all completed questionnaires on a daily basis to ensure data completeness and consistency. Prior to analysis, the dataset was thoroughly checked and cleaned to address any inconsistencies or missing information.

### 2.4 Assessment of Dietary Intake

A 24‐h dietary recall method was implemented to collect dietary intake data from RMG workers. Visual assistance, such as food pictures and diagrams, was provided to help the respondents recall the food items and portion sizes efficiently. Portion sizes were determined according to the descriptions of respondents, where visual aids could not be provided. As data collection was performed in the workplace, the quantity of food was measured by cups, spoons, and food models. The staple rice intake was measured carefully. Conversion factors were applied where the raw weight of ingredients was not available. Food items reported in grams or milliliters as consumption units were included, whereas those reported in cups or numbers were converted into equivalent gram units. The consumption unit for eggs, reported as the number of pieces, was converted into grams by multiplying the number by its average portion size (50 g). Additionally, incorrectly coded food items were removed from the dataset. Finally, a total of 254 food items were extracted for the analysis.

### 2.5 Dietary Pattern Analysis

All food items were categorized into 18 food groups (Table S1) for factor analysis to determine dietary patterns. These groups were created on the basis of the participants’ typically consumed food items and the shared nutritional content of the items [35]. PCA was conducted on the daily intakes of 18 food groups, measured in grams/milliliters, for individuals residing in both the Dhaka and Chattogram regions. The orthogonal (Varimax) rotation method was employed to derive uncorrelated factors for enhanced interpretability [39]. The determination of the number of factors was based on eigenvalues >1.5, serving as a cutoff criterion for retaining factors. This decision was informed by evaluating eigenvalues, assessing the scree plot, and conducting an interpretability analysis [40]. The *p* value of Barlett's test of sphericity was <0.001, and the Kaiser–Meyer–Olkin (KMO) measure of sampling adequacy was 0.67, indicating that the variables were deemed suitable for factor analysis [41].

Individual food groups with a factor loading ≥0.3 were considered to significantly contribute to the pattern in this study [42]. The labeling of dietary patterns was based on the interpretation of foods with high factor loadings for each dietary pattern [43]. Then the factor score was derived by using the regression method, which provided a score for each factor [41]. The score indicates the adherence of the participants to that dietary pattern. Finally, factor scores of each dietary pattern of the corresponding participants were categorized into terciles, where *T*1 indicated the lowest and *T*3 indicated the highest adherence or intake of each dietary pattern.

### 2.6 Assessment of Dietary Adequacy

In this study, we utilized the Bangladeshi Food Composition Table (FCT) to accurately calculate the energy and nutrient intake of the participants [44]. The adequacy of macronutrient intake was assessed by comparing the percentage of energy derived from three macronutrients to the Acceptable Macronutrient Distribution Ranges (AMDR) [45]. The mean adequacy ratio (MAR) was constructed using the nutrient adequacy ratio (NAR). The NAR was assessed using the age‐ and sex‐specific estimated average requirement (EAR) values for micronutrients [45]. The NAR of different micronutrients was calculated as Equation (1). When the NAR value was greater than 1, it was truncated to 1. MAR was obtained by averaging all of the reduced NAR values Equation (2) [46]. Thus, the MAR was expressed on a scale of 0 to 1, with 0 denoting that no nutrient requirement was met and 1 denoting that all nutrient requirements were met:

(1) $$\begin{array}{ccc} & & \mathrm{Nutrient}\;\mathrm{adequacy}\;\mathrm{ratio}\;\left(\mathrm{NAR}\right)\\ & = & \frac{\mathrm{Daily}\;\mathrm{nutrient}\;\mathrm{intake}}{\mathrm{Estimated}\;\mathrm{Average}\;\mathrm{Requirement}\;\left(\mathrm{EAR}\right)} \end{array}$$

(2) $$\mathrm{Mean}\;\mathrm{adequacy}\;\mathrm{ratio}\;\left(\mathrm{MAR}\right)=\frac{\sum \mathrm{NAR}\;\left(\mathrm{Each}\;\mathrm{truncated}\;\mathrm{at}\;1\right)}{\mathrm{Number}\;\mathrm{of}\;\mathrm{nutrients}}$$

### 2.7 Assessment of Body Composition

The body fat percentages of the respondents were assessed through the bioelectrical impedance analyzer (BIA) technique using the Tanita scale, Model UM 070 [47]. BIA involves measuring the impedance, or resistance, encountered by a low‐level electrical current as it passes through the body. Lean tissue, which has a higher water content, conducts electricity more effectively than fat tissue, which has less water. In a BIA measurement, electrodes are positioned on the participant's skin, typically on the hand, foot, wrist, or ankle. A mild electrical current is then passed through the body, and the resulting impedance or resistance is recorded. The BIA device employs mathematical equations that often consider factors such as age, gender, height, and weight to estimate body composition. It calculates the ratio of fat to lean mass based on electrical impedance. Body fat percentage was categorized using sex‐specific cut‐off values as follows: for men, low/normal (<25%) and high (≥25%); and for women, low/normal (<30%) and high (≥30%) [48].

### 2.8 Assessment of Nutritional Status

The height of each participant was measured by a portable stadiometer (height scale), and the weight of the respondents was measured by a portable electronic scale (Tanita weight scale, HA‐650, Japan). BMI was calculated by dividing the weight in kilograms by the square of the height in meters (kg/m^2^). Nutritional status (BMI) categories were determined on the basis of the Asian‐specific BMI cut‐off [49]: underweight (BMI < 18.5 kg/m^2^), normal weight (BMI 18.5–22.9 kg/m^2^), overweight (BMI 23 to <27.5 kg/m^2^), and obese (BMI ≥ 27.5 kg/m^2^).

### 2.9 Statistical Analysis

The study variables were presented as frequency, percentage, mean, SD, median, and IQR. Additionally, chi‐square test was conducted for the categorical variables to ascertain the association of covariates on body fat percentage. Moreover, the association between body fat percentage and dietary patterns was evaluated using bivariate and multivariate logistic regression. In the multivariate model, we adjusted for all potential covariates with *p* values <0.25 in the bivariate model [50]. These covariates included age (≤23, 24, 25, 25–28, >28 years), sex (male, female), educational status (up to primary, up to secondary, higher secondary, and above), marital status (married, unmarried), household income in BDT (low: ≤20,000, medium: 20,001–26,000, high: >26,000), and food expenditure in BDT (<6000, 6000–10,000, >10,000). The Hosmer–Lemeshow goodness‐of‐fit test was used to evaluate the fit of the regression model. Multicollinearity among the independent variables was evaluated using the variance inflation factor (VIF), with a threshold of VIF <2 indicating no significant collinearity [51]. The model fit assumptions for the logistic regression analysis are presented in Tables S2 and S3. The results of this study were reported as both the crude odds ratios (CORs) and adjusted odds ratios (AORs) with 95% confidence intervals (CIs). A *p* value of <0.05 was considered statistically significant. These statistical analyses were carried out in SPSS (version 26.0) for Windows.

## 3 Results

Table 1 presents the socioeconomic characteristics of study participants from Dhaka and Chattogram. A majority of the participants were females (60.9%), with a slightly higher proportion in Chattogram (62.6%) compared to Dhaka (59.8%). The largest age group was 23 years or younger (36.1%), with Chattogram having a greater concentration of younger participants (40.0%) compared to Dhaka (33.5%). Educational attainment was fairly consistent across both cities, with 46.0% of participants having up to secondary education. Most of the participants were married (75.7%), particularly in Dhaka, where 79.8% of respondents reported being married. Living arrangements differed significantly between the two cities; overall, 53.6% of participants were renting, with all participants in Chattogram (100%) living in rented accommodations. Regarding household income, 41.8% of the participants reported earning ≤BDT 20,000 per month, and the majority allocated between BDT 6000 and 10,000 to food expenditures.

**TABLE 1:** Socioeconomic characteristics of the study participants (*N* = 576).

Characteristics	Overall (*N* = 576) *n* (%)	Dhaka (*N* = 346) *n* (%)	Chattogram (*N* = 230) *n* (%)
**Sex**			
Male	225 (39.1)	139 (40.2)	86 (37.4)
Female	351 (60.9)	207 (59.8)	144 (62.6)
**Age (years)**			
≤23	208 (36.1)	116 (33.5)	92 (40.0)
24–25	91 (15.8)	56 (16.3)	35 (15.2)
26–28	136 (23.6)	87 (25.1)	49 (21.3)
>28	141 (24.5)	87 (25.1)	54 (23.5)
**Educational status**			
Up‐to primary	90 (15.6)	50 (14.5)	40 (17.4)
Up‐to secondary	265 (46.0)	158 (45.7)	107 (46.5)
Higher secondary and above	221 (38.4)	138 (39.9)	83 (36.1)
**Marital status**			
Unmarried	140 (24.3)	70 (20.2)	70 (30.4)
Married	436 (75.7)	276 (79.8)	160 (69.6)
**Living status**			
In own household	267 (46.4)	267 (77.2)	0 (0.0)
Rent	309 (53.6)	79 (22.8)	230 (100.0)
**Household monthly income (BDT)**			
Low (≤20,000)	241 (41.8)	156 (45.1)	85 (37.0)
Medium (20,001–26,000)	144 (25.0)	89 (25.7)	55 (23.9)
High (>26,000)	191 (33.2)	101 (29.2)	90 (39.1)
**Monthly food expenditure (BDT)**			
<6000	226 (39.2)	146 (42.2)	80 (34.8)
6000–10,000	239 (41.5)	141 (40.8)	98 (42.6)
>10,000	111 (19.3)	59 (17.1)	52 (22.6)

*Note:* $1 = 121.5 BDT.

Abbreviation: BDT, Bangladeshi taka.

Overall, 8.7% of participants were underweight, with a higher proportion in Chattogram (11.3%) compared to Dhaka (6.9%). About 40% of the participants had normal weight, followed by 39.4% who were overweight and 11.8% classified as obese. Regarding body fat percentages, 49.1% had normal fat levels, whereas 33.0% had high fat and 14.2% had very high fat (Table 2).

**TABLE 2:** Distribution of nutritional status and body fat percentage among study participants.

Characteristics	Overall (*N* = 576) *n* (%)	Dhaka (*N* = 346) *n* (%)	Chattogram (*N* = 230) *n* (%)	*p* value
**Nutritional status**				
Underweight (BMI < 18.5 kg/m^2^)	50 (8.7)	24 (6.9)	26 (11.3)	0.191
Normal (BMI 18.5–22.9 kg/m^2^)	231 (40.1)	135 (39.0)	96 (41.7)	
Overweight (BMI 23 to <27.5 kg/m^2^)	227 (39.4)	145 (41.9)	82 (35.7)	
Obese (BMI ≥ 27.5 kg/m^2^)	68 (11.8)	42 (12.1)	26 (11.3)	
**Body fat percentage category**				
Low/Normal fat	317 (55.0)	182 (52.6)	135 (58.7)	0.088
High fat	259 (45.0)	164 (47.4)	95 (41.3)	

*Note: p* value from Pearson chi‐square test, the cut‐off values used to classify body fat percentage are detailed in Table 1.

Abbreviation: BMI, body mass index.

The daily nutrient intake of the study participants, as presented in Table 3, shows varying levels of adherence to recommended dietary intake guidelines. Median energy intake was 2049 kcal. Carbohydrate intake had a median of 330.4 g, with 1.9% of participants consuming below the reference and 47.6% above it. Additionally, carbohydrates appeared to be the primary source of energy, constituting approximately 67.7% of the total energy intake (Table S4). Notably, 20.5% consumed below the fat reference range. Micronutrient intake exhibited considerable deficiencies, with 88.9% of participants consuming below the reference for calcium, 80% for iron, 76.7% for vitamin A, 73.1% for zinc, and 90.8% for riboflavin. The NAR for several nutrients, including calcium and vitamin A, was particularly low (0.33 and 0.50, respectively). However, intake for niacin and folate was above the reference for 80% and 55.6% of participants, respectively, with relatively higher NARs. Across all 11 micronutrients, the MAR value was calculated at 0.72.

**TABLE 3:** Daily intake of nutrients by the study participants.

Nutrients	Median (IQR)	Daily intake by the respondents according to reference intake		NAR mean	MAR mean (SD)
		Below reference *n* (%)	Above reference *n* (%)		
Energy (kcal)	2049 (1779, 2443)	—	—	—	0.72 (0.15)
Carbohydrate (g)	330.4 (279.9, 398.6)	11 (1.9)	274 (47.6)	—	
Protein (g)	65.1 (51.5, 80.9)	0.0	121 (21.0)	—	
Fat (g)	46.7 (35.3, 59.1)	118 (20.5)	28 (4.9)	—	
Vitamin A (mcg)	205 (110, 406.5)	442 (76.7)	134 (23.3)	0.50	
Thiamine (mg)	1.1 (0.9, 1.5)	281 (48.8)	295 (51.2)	1.01	
Riboflavin (mg)	0.8 (0.6, 1.1)	523 (90.8)	53 (9.2)	0.50	
Niacin (mg)	15.8 (11.4, 21.0)	115 (20.0)	461 (80.0)	1.60	
Vitamin B6 (mg)	1.5 (1.2, 1.9)	322 (55.9)	254 (44.1)	0.94	
Folate (mcg)	226 (138, 651.2)	256 (44.4)	320 (55.6)	1.13	
Vitamin B12 (mcg)	1.34 (0.5, 2.6)	363 (63.0)	211 (36.6)	0.67	
Vitamin C (mg)	57.2 (28.6, 106.4)	286 (49.7)	290 (50.3)	1.00	
Calcium (mg)	265 (140, 517)	512 (88.9)	64 (11.1)	0.33	
Iron (mg)	8.4 (6.5, 12.0)	461 (80.0)	115 (20.0)	0.65	
Zinc (mg)	9.9 (8.2, 12.3)	421 (73.1)	155 (26.9)	0.82	

*Note:* The adequacy of macronutrient intake was assessed by comparing the percentage of energy derived from three macronutrients to the AMDR established by the ICMR (2020), AMDR values, carbohydrate (45%–65%), protein (5%–15%), and fat (15%–35%).

Abbreviations: AMDR, acceptable macronutrient distribution ranges; MAR, mean adequacy ratio; NAR, nutrient adequacy ratio.

Table 4 presents the factor loading matrix of the identified total of five dietary patterns through PCA among the RMG workers living in the Dhaka and Chattogram regions. Two dietary patterns were identified among RMG workers living in the Dhaka region, which explained cumulatively 17.4% of the total dietary intake variance. The first pattern, termed the “typical Bangladeshi diet,” explained 9.1% of the total variance and was characterized by high factor loadings for rice, fish, leafy vegetables, oils, and spices. The second pattern, labeled the “fruits and fast food diet,” explained 8.3% of the variance and was characterized by a higher consumption of fruits, processed cereals, and fast foods. In the Chattogram region, three dietary patterns were identified, cumulatively explaining 30.4% of the total dietary intake variance. The first and most dominant pattern was the “dairy‐based festival diet,” which explained 11.9% of the variance. This pattern was characterized by high intakes of dairy products (milk, yogurt), sweets, processed cereals (vermicelli, semolina, bread, cake), fast foods, and wheat‐based foods. Food items loading strongly on this pattern included vermicelli, semolina, cake, yogurt, confections, payesh (traditional rice pudding), ruti (flatbread), noodles, and beguni (fried snack). The second pattern identified in Chattogram was the “vegetables and fish‐based diet,” which explained 9.6% of the total variance and was characterized by high loadings of a diverse range of starchy and non‐starchy vegetables along with various types of fish. The third pattern was the “typical Bangladeshi diet,” explaining 8.9% of the variance, and was characterized by higher intakes of rice, vegetables, pulses/legumes, and meat.

**TABLE 4:** Factor‐loading matrix for the dietary patterns of the ready‐made garment (RMG) workers living in Dhaka and Chattogram.

	Dietary patterns in Dhaka		Dietary patterns in Chattogram		
Food groups	Typical Bangladeshi diet	Fruits and fast food‐based diet	Dairy‐based festival diet	Vegetables and fish‐based diet	Typical Bangladeshi diet
Rice	0.58	—	—	—	0.34
Wheat flour	—	—	0.34	0.31	—
Other processed cereals	—	0.44	0.41	—	—
Starchy vegetables	—	—	—	0.52	—
Non‐starchy vegetables	—	—	—	0.50	—
Leafy vegetables	0.34	—	—	—	0.62
Pulse/Legumes	—	—	—	—	0.59
Nuts/Seeds		—	—	—	—
Fruits		0.58	—	—	—
Meat	—	—	—	—	0.38
Fish	0.44	—	—	0.67	—
Egg	—	—	—	—	—
Dairy	—	—	0.45	—	—
Fast foods	—	0.57	0.49	—	—
Sweets	—	—	0.45	—	—
Oils	0.44	—	0.61	0.32	—
Spices	0.48	—	0.60	0.39	—
Beverages	—	—	—	—	—
Variance of intake explained (%)	**9.1**	**8.3**	**11.9**	**9.6**	**8.9**

*Note:* Factor loadings ≥0.3 were considered to significantly contribute to the pattern in this study.

The associations between dietary patterns and body fat percentage are shown in Table 5. Regarding both the “typical Bangladeshi diet” and “fruits and fast food‐based diet” in the Dhaka region, the odds ratios (ORs) for higher body fat percentage compared to the reference group (*T*1) were not statistically significant across all terciles (*T*2 and *T*3). But in the Chattogram region, the results indicate that for the “dairy‐based festival diet,” individuals in the highest tercile had significantly higher odds of having a higher body fat percentage compared to those in the lowest tercile (AOR = 2.44, 95% CI: 1.41, 5.75; *p* = 0.040). However, no significant associations were observed between body fat percentage and the “vegetables and fish‐based diet” or the “typical Bangladeshi diet” in this region. These findings remained consistent after adjusting for potential confounding variables such as sex, age, education, marital status, income, and food expenditure.

**TABLE 5:** Association between dietary patterns and body fat percentage of the ready‐made garment (RMG) workers living in Dhaka and Chattogram region.

Variables	Higher body fat percentage			
	COR (95% CI)	*p* value	AOR (95% CI)	*p* value
**Dhaka region**				
**Typical Bangladeshi diet**				
*T*1 (lowest)	1 (Ref.)	—	1 (Ref.)	—
*T*2	1.07 (0.67, 1.81)	0.689	1.32 (0.65, 2.32)	0.524
*T*3 (highest)	1.04 (0.61, 1.77)	0.863	1.11 (0.58, 1.78)	0.724
**Fruits and fast food‐based diet**				
*T*1 (lowest)	1 (Ref.)	—	1 (Ref.)	—
*T*2	0.66 (0.38, 1.14)	0.138	0.85 (0.40, 1.79)	0.670
*T*3 (highest)	0.56 (0.38, 1.06)	0.065	0.54 (0.26, 1.26)	0.095
**Chattogram region**				
**Dairy‐based festival diet**				
*T*1 (lowest)	1 (Ref.)	—	1 (Ref.)	—
*T*2	1.21 (0.47, 2.13)	0.508	2.04 (0.89, 4.47)	0.090
*T*3 (highest)	2.19 (1.12, 3.42)	0.023	2.44 (1.41, 5.75)	0.040
**Vegetables and fish‐based diet**				
*T*1 (lowest)	1 (Ref.)	—	1 (Ref.)	—
*T*2	1.34 (0.71, 2.51)	0.357	1.25 (0.85, 3.32)	0.650
*T*3 (highest)	1.45 (0.76, 2.79)	0.256	1.92 (0.98, 2.89)	0.069
**Typical Bangladeshi diet**				
*T*1 (lowest)	1 (Ref.)	—	1 (Ref.)	—
*T*2	0.77 (0.41, 1.44)	0.426	0.60 (0.27, 1.34)	0.219
*T*3 (highest)	1.30 (0.67, 2.52)	0.423	1.03 (0.44, 2.40)	0.932

*Note: T* indicates the tercile of loading factors of each dietary pattern. Adjusted for sex, age, education, marital status, household income, and food expenditure.

Abbreviations: AOR = adjusted odds ratio; CI = confidence interval; COR = crude odds ratio; Ref. = reference.

## 4 Discussion

This study examined the dietary habits, body composition, and nutritional status of RMG workers, as well as the relationship between dietary patterns and body composition. The findings revealed a high prevalence of overweight/obesity and elevated body fat levels among the workers. Carbohydrates emerged as the primary energy source, contributing to 67.7% of total energy, and deficiencies in essential micronutrients were prevalent, with the MAR value of 0.72. A total of five major dietary patterns were identified across the two regions. Notably, in the Chattogram region, workers who adhered more closely to the “dairy‐based festival diet” exhibited a significantly higher body fat percentage.

In this study, nearly half of the RMG workers exhibited a higher body fat percentage, and approximately half were classified as overweight or obese. These findings align with previous research among RMG workers in Bangladesh, where 44% were reported as overweight or obese based on BMI [52]. Another study also documented a comparable prevalence, with 50.4% of garment workers classified as overweight [53]. The high prevalence of excess body fat and overweight/obesity among RMG workers may be attributed, in part, to the sedentary nature of their work, especially among female workers, as highlighted in earlier studies [53]. Additionally, the dietary habits of this population may contribute to their nutritional imbalance, suggesting that both occupational and lifestyle factors play a significant role in shaping their body composition [52].

Consistent with earlier research, this study revealed an imbalanced macronutrient intake among participants, characterized by a tendency toward excessive carbohydrate consumption and inadequate fat intake relative to dietary recommendations [52]. In line with this, another study found that cereals—particularly rice—constitute the primary component of the diet among garment workers, accounting for 52.37% of total food consumption [37]. This could be attributed to low‐income levels and limited nutritional knowledge, as workers in the RMG sector in Bangladesh generally belong to lower income groups [54] and often have insufficient knowledge of nutrition [55]. A previous study showed a linear relationship between higher income and increased intake of fat and protein, whereas carbohydrate consumption tends to decrease with rising income [56]; our findings are consistent with this.

Our study found a MAR value for micronutrients of 0.72, alongside notable inadequacies in key micronutrients such as calcium, iron, vitamin A, and riboflavin. These findings are aligned with a previous study, which reported that over half of RMG workers had inadequate micronutrient intake, reflected by a mean probability of adequacy (MPA) score below 0.5 and intakes of calcium, iron, zinc, riboflavin, and vitamin A falling below the EARs, indicating a high risk of micronutrient deficiencies [52]. These inadequacies may be attributed to the RMG workers’ adherence to a less diversified diet, as reported in a previous study [37], which found that the workers had a limited variety of food in their diet, with cereals, particularly rice, being the main component, while the consumption of dairy, eggs, meat, vegetables, fish, pulses, and edible oil was insufficient. A number of studies across different populations have indicated that there is a correlation between a lower diversity of food and a higher risk of micronutrient deficiencies [57, 58].

In our study, the typical Bangladeshi pattern was highly loaded with rice, fish, leafy vegetables, oils, and spices. In contrast, the fruits and fast food pattern was characterized by a higher consumption of fruits, processed cereals, and fast foods. Although the “typical Bangladeshi diet” was also common in Chattogram, two additional dietary patterns were also observed, namely, “dairy‐based festival diet” and “vegetables and fish‐based diet.” The pattern “dairy‐based festival diet” was highly loaded with fast food, processed cereals, and sweets, predominantly comprising milk and sugar derivatives. This pattern includes food items traditionally prepared and consumed intermittently, particularly during festive occasions, such as vermicelli, semolina, cake, yogurt, confections, payesh (a traditional rice pudding), ruti (flatbread), noodles, and beguni (a fried snack). The second pattern, termed the “vegetables and fish‐based diet,” incorporates a diverse array of starchy and non‐starchy vegetables along with various types of fish. Due to its proximity to the Bay of Bengal, Chattogram's residents commonly include fish as a significant component of their daily diet, with the region leading Bangladesh in annual fish consumption [59]. This dietary habit reflects both cultural preferences and the availability of fresh seafood in the local area. The third dietary pattern identified among workers residing in the Chattogram region is referred to as the “typical Bangladeshi diet.” This diet primarily includes rice, vegetables, pulses/legumes, and meat. Such a pattern mirrors the traditional dietary practices observed in Bangladesh [32], highlighting the cultural significance of these food choices within the local community.

Previous studies on dietary patterns in Bangladesh have revealed various patterns. A study conducted in Bangladesh identified three major dietary patterns: a balanced diet, an animal protein diet, and a root vegetable diet [33]. Similarly, another study highlighted three distinct dietary patterns: balanced diet, gourd vegetable diet, and Western diet [34]. Both studies utilized PCA to discern these patterns. In a separate investigation, seven distinct patterns were identified using PCA [39]. The basic components of the patterns identified in the previous studies included rice, vegetables, pulses, fish, meats, spices, and oils, which are similar to the foods that make up the patterns identified in the present study—for instance, the three patterns described by Chen et al. [33] were “balanced diet,” characterized by rice, fish, meat, vegetables, and fruits; “animal protein diet,” and “gourd and root vegetable diet,” highly loaded with different types of vegetables. The dietary patterns referred to as the “balanced diet” and the “gourd and root vegetable diet” in the previous study are nearly identical to the dietary patterns identified as the “typical Bangladeshi diet” and the “vegetables and fish‐based diet,” respectively, as highlighted in the current study.

In the present study, in the Chattogram region, one dietary pattern showed a significant association with body fat percentage. Specifically, the “dairy‐based festival diet” significantly increased the risk of higher body fat by 2.15 times. The majority of the foods in the “dairy‐based festival diet” are high in carbohydrates, sugars, and fats, which can contribute to increased calorie intake and subsequent weight gain if consumed in excess. For instance, vermicelli is often used in sweet dishes and can be high in simple carbohydrates and sugars. Similarly, semolina, a refined grain product, can be calorie‐dense and high in carbohydrates. Moreover, cakes are typically high in sugar and fats, contributing to high‐calorie content. Although yogurt can be healthy in moderation, some varieties are high in added sugar. Additionally, sweets are usually high in sugars and fats, leading to high‐calorie intake. Payesh contains sugar and sometimes high‐fat milk or cream, making it calorie‐rich. Furthermore, noodles are often high in refined carbohydrates and can be calorie‐dense, especially if fried or served with high‐fat sauces. Beguni, being typically fried, adds significant amounts of fats and calories. Therefore, regular consumption of these items, especially in large quantities, can contribute to a higher overall calorie intake and potentially increase body fat. A study reported that refined grain products, such as vermicelli and semolina, are calorie‐dense and high in carbohydrates. Consequently, consuming these refined grains is associated with weight gain and an increased body fat percentage [60]. Similarly, according to another study, cakes are typically high in sugar and fat, contributing to a high calorie content, which can also lead to weight gain [61]. Moreover, a different study stated that sweets and confections are high in sugars and fats, leading to high calorie intake, which can increase body fat when consumed excessively [62]. Therefore, our study results are supported by similar findings from previous research.

To address the high prevalence of overweight/obesity, elevated body fat levels, and micronutrient deficiencies among RMG workers, several targeted initiatives are recommended. Factory authorities can play a pivotal role in improving workers’ nutritional well‐being by implementing targeted nutrition education programs that promote healthier eating habits. Establishing fair‐price shops that offer affordable, nutrient‐rich foods, alongside subsidized meal programs and upgraded workplace canteens, can significantly enhance access to balanced diets. These initiatives can help reduce excessive carbohydrate consumption while increasing the intake of protein‐ and micronutrient‐rich foods. Furthermore, introducing weight management counseling can support workers in adopting and maintaining healthier lifestyles. Evidence suggests that interventions such as nutrition education to enhance the desirability of healthy diets, the establishment of Fair Price Shops and nutrition corners in factory canteens, and the provision of midday meals—implemented by the Global Alliance for Improved Nutrition, Bangladesh—have significantly improved the nutritional well‐being of workers [63]. At the government level, policies should integrate nutrition into labor welfare programs by ensuring regular health screenings and dietary counseling for workers. Offering subsidies or tax incentives can encourage factories to implement workplace nutrition initiatives. Enforcing food quality standards in factory canteens and promoting public–private partnerships can also improve access to affordable, nutritious foods. Collectively, these measures can enhance worker health, well‐being, and productivity, supporting the long‐term sustainability of the RMG sector.

### 4.1 Strengths and Limitations

To our knowledge, this is the first study to explore the relationship between dietary patterns and body fat percentage among RMG workers in Bangladesh, conducted in Dhaka and Chattogram, prominent centers of the country's RMG sector. The study addresses various critical aspects of RMG workers’ health and nutrition, and its findings could inform policy decisions aimed at improving working conditions, labor rights, and the overall welfare of these workers, ultimately promoting positive social and economic transformations.

Due to institutional regulations and the unavailability of a complete worker roster, random sampling was not feasible, and issues of representativeness and selection bias may have arisen. However, we have tried to mitigate this by including workers from all floors, all job sections, and both genders. The measurement of body fat percentage using the BIA method can be affected by individual differences in hydration status. To address this, individual hydration status was considered, and the BIA scale was routinely calibrated. Dietary data were collected using the 24‐h recall method, which is susceptible to recall bias. To minimize this, enumerators underwent rigorous training, employed the reliable AMPM technique, and used food item show cards to enhance data accuracy.

## 5 Conclusion

This study highlights the high prevalence of overweight, obesity, and elevated body fat among RMG workers, alongside a carbohydrate‐dominant diet and widespread micronutrient deficiencies. Notably, greater adherence to a “dairy‐based festival diet” was linked to increased body fat percentage in the Chattogram region. These findings emphasize the need for targeted nutritional interventions to address rising obesity rates and improve diet quality. Workplace nutrition programs, dietary education, and policy‐driven initiatives can enhance workers' health, productivity, and overall well‐being. Further research is needed to establish causal links between dietary patterns and body composition and to develop effective strategies for improving workforce nutrition.

## Author Contributions

Md. Ruhul Amin conceptualized the study. Md. Ruhul Amin and Md. Shahadoth Hossain designed the analytical approach. Md. Shahadoth Hossain performed the data analysis. Md. Shahadoth Hossain, Md. Hafizul Islam, Rafid Hassan, Md. Mahbub Alam, and G. M. Reza Sumon contributed to the interpretation of the findings. Md. Shahadoth Hossain, Md. Hafizul Islam, and Rafid Hassan drafted the initial manuscript. Md. Ruhul Amin supervised the study. All authors critically reviewed, provided substantial input, and approved the final manuscript. Md. Ruhul Amin is the guarantor of the study.

## Funding

The authors have nothing to report.

## Ethics Statement

The Institutional Review Board of the Faculty of Biological Sciences, University of Dhaka, has thoroughly reviewed and approved the study (Ref. No. 223/Biol. Scs). The nature and purpose of the study were thoroughly explained to all participants, and their informed oral consent was obtained prior to the commencement of the study.

## Conflicts of Interest

The authors declare no conflicts of interest.

## Supporting information



Supporting file 1: puh270195‐sup‐0001‐SuppMat.docx

## Data Availability

The data will be available from the corresponding author on reasonable request.
