# Supplementary material for: Dietary Patterns and Their Association With Body Fat Percentage Among Ready‐Made Garment Workers in Dhaka and Chattogram, Bangladesh
Source: Public Health Chall. 2026 Feb 13;5(1):e70195. doi: 10.1002/puh2.70195 (PMC12904289; doi:10.1002/puh2.70195)
Supplement: Supplementary file 1 — Supporting file 1: puh270195‐sup‐0001‐SuppMat.docx [file PUH2-5-e70195-s001.docx]

**Supplementary Tables**:

**S. Table 1.** Food items and food groupings

| **Food groups** | **Food items** |
| --- | --- |
| Rice | Rice coarse, Rice medium, Rice fine |
| Wheat or flour | Wheat (Atta), Flour |
| Other processed cereals | Puffed rice, Bread, Vermicelli, Semolina, Cake, Biscuits |
| Starchy vegetables | Potato, Arum / Ol kochu, Kochur mukhi |
| Non starchy vegetables | Tomato, Brinjal, White gourd / pumpkin, Green banana, |
| Leafy vegetables | All types of leafy vegetables (Spinach /Amaranth / Basil) |
| Pulse & legumes | Lentil, Bengal gram, Green gram, Black gram |
| Nuts and seeds | Almond, Peanut, Jackfruits seed, Pumpkin seed |
| Fruits | Ripe banana, Mango, Guava, Apple, Malta |
| Meat | Chicken, Beef, Mutton |
| Fish | Telapia, Rhui / Catla / Mrigel, Pangash, Silver carp |
| Eggs | Eggs (Chicken & Duck) |
| Dairy | Whole milk, Yoghurt, Cheese |
| Fast foods/Fried foods | Shingara, Beguni, Puri, Noodles |
| Sweets | Sugar, Molasses, Honey, Jilapi |
| Oils | Soybean oil, Mustard oil, Palm oil |
| Spices and condiments | All types of spices and condiments |
| Beverages | Tea, coffee, Juices and drinks |

**S. Table 2.** Model fit assumptions for logistic regression analysis are provided in Supplementary Table 3 and 4) (Model 1: Dhaka region)

| **Factors** | **Collinearity Statistics** | | **Hosmer and Lemeshow test** | **Nagelkerke R Square** | **Omnibus test** |
| --- | --- | --- | --- | --- | --- |
|  | **Tolerance** | **VIF** |  |  |  |
| Dietary pattern 1 | 0.983 | 1.018 | 0.951 | 0.211 | < 0.001 |
| Dietary pattern 2 | 0.977 | 1.017 |  |  |  |
| Age | 0.857 | 1.117 |  |  |  |
| Sex | 0.762 | 1.275 |  |  |  |
| Marital status | 0.891 | 1.085 |  |  |  |
| Educational status | 0.849 | 1.276 |  |  |  |
| Monthly income | 0.754 | 1.234 |  |  |  |
| Monthly food expenditure | 0.781 | 1.374 |  |  |  |

**S. Table 3.** Model fit assumptions for logistic regression analysis (Model 2: Chattogram region)

| **Factors** | **Collinearity Statistics** | | **Hosmer and Lemeshow test** | **Nagelkerke R Square** | **Omnibus test** |
| --- | --- | --- | --- | --- | --- |
|  | **Tolerance** | **VIF** |  |  |  |
| Dietary pattern 1 | 0.963 | 1.049 | 0.231 | 0.221 | < 0.001 |
| Dietary pattern 2 | 0.987 | 1.055 |  |  |  |
| Dietary pattern 3 | 0.937 | 1.065 |  |  |  |
| Age | 0.829 | 1.218 |  |  |  |
| Sex | 0.864 | 1.205 |  |  |  |
| Marital status | 0.915 | 1.105 |  |  |  |
| Educational status | 0.852 | 1.190 |  |  |  |
| Monthly income | 0.694 | 1.496 |  |  |  |
| Monthly food expenditure | 0.688 | 1.475 |  |  |  |

**S. Table 4.** Distribution of energy contribution from macronutrients consumed by the study participants

| **Macronutrients** | **Overall (n = 576)** | **Dhaka (n=346)** | **Chattogram (n=230)** |
| --- | --- | --- | --- |
| Carbohydrate (%) | 67.7 | 67.7 | 67.8 |
| Protein (%) | 12.4 | 12.3 | 12.5 |
| Fat (%) | 19.9 | 20 | 19.7 |
